# Review and Comparative Evaluation of Mobile Apps for Cardiovascular Risk Estimation: Usability Evaluation Using mHealth App Usability Questionnaire

**DOI:** 10.2196/56466

**Published:** 2025-05-08

**Authors:** Adrijana Svenšek, Lucija Gosak, Mateja Lorber, Gregor Štiglic, Nino Fijačko

**Affiliations:** 1 Faculty of Health Sciences, University of Maribor, Žitna ulica 15, Maribor, 2000, Slovenia, 386 2 300 4762; 2 Faculty of Electrical Engineering and Computer Science, University of Maribor, Maribor, Slovenia; 3 Usher Institute, University of Edinburgh, Edinburgh, United Kingdom; 4 University Medical Centre, Maribor, Slovenia

**Keywords:** cardiovascular diseases, MAUQ, prognostic models, mobile applications, visualization, PRISMA

## Abstract

**Background:**

Cardiovascular diseases (CVD) are the leading cause of death and disability worldwide, and their prevention is a major public health priority. Detecting health issues early and assessing risk levels can significantly improve the chances of reducing mortality. Mobile apps can help estimate and manage CVD risks by providing users with personalized feedback, education, and motivation. Incorporating visual analysis into apps is an effective method for educating society. However, the usability evaluation and inclusion of visualization of these apps are often unclear and variable.

**Objective:**

The primary objective of this study is to review and compare the usability of existing apps designed to estimate CVD risk using the mHealth App Usability Questionnaire (MAUQ). This is not a traditional usability study involving user interaction design, but rather an assessment of how effectively these applications meet usability standards as defined by the MAUQ.

**Methods:**

First, we used predefined criteria to review 16 out of 2238 apps to estimate CVD risk in the Google Play Store and the Apple App Store. Based on the apps’ characteristics (ie, developed for health care professionals or patient use) and their functions (single or multiple CVD risk calculators), we conducted a descriptive analysis. Then we also compared the usability of existing apps using the MAUQ and calculated the agreement among 3 expert raters.

**Results:**

Most apps used the Framingham Risk Score (8/16, 50%) and Atherosclerotic Cardiovascular Disease Risk (7/16, 44%) prognostic models to estimate CVD risk. The app with the highest overall MAUQ score was the MDCalc Medical Calculator (mean 6.76, SD 0.25), and the lowest overall MAUQ score was obtained for the CardioRisk Calculator (mean 3.96, SD 0.21). The app with the highest overall MAUQ score in the “ease-of-use” domain was the MDCalc Medical Calculator (mean 7, SD 0); in the domain “interface and satisfaction,” it was the MDCalc Medical Calculator (mean 6.67, SD 0.33); and in the domain “usefulness,” it was the ASCVD Risk Estimator Plus (mean 6.80, SD 0.32).

**Conclusions:**

We found that the Framingham Risk Score is the most widely used prognostic model in apps for estimating CVD risk. The “ease-of-use” domain received the highest ratings. While more than half of the apps were suitable for both health care professionals and patients, only a few offered sophisticated visualizations for assessing CVD risk. Less than a quarter of the apps included visualizations, and those that did were single calculators. Our analysis of apps showed that they are an appropriate tool for estimating CVD risk.

## Introduction

The major cause of death worldwide is cardiovascular diseases (CVD). The prevalence of CVDs has increased globally due to factors such as obesity, poor nutrition, hypertension, and the incidence of type 2 diabetes [1,2]. CVDs still affect more than half a billion people worldwide, causing 20.5 million deaths in 2021, which is almost a third of all deaths worldwide [3]. The approach to preventing CVD morbidity is to reduce and control risk factors through lifestyle changes and medication. Early detection and risk stratification further augment the chances of reducing mortality [4,5]. Many prognostic models have been developed in recent decades that assess the risk of CVDs [6,7], for example, the Framingham Heart Score for the American population [8,9], SCORE2 (Systematic Coronary Risk Evaluation 2) for European populations [10], QRISK (Cardiovascular Risk Score) for the British population [11-13], and artificial intelligence (AI)-based prediction model’s [14] and others.

The rise of IT, such as mobile health (mHealth) tools, can make health care more accessible because of the integration of many prognostic models. It has been shown they can be used to reduce the risk of CVDs [6,15-17], improve glycemic control [18,19], control elevated blood pressure [20,21], contribute to the reduction in hospital admissions as part of cardiac rehabilitation [17,22], and also eHealth information exchange could give patients, who are from rural areas or have mobility problems, access to their health data – including the ability to capture and aggregate multiple sources of clinical data for performance measurement [23,24].

Growing research evidence supports the effectiveness of mobile apps for the prevention of CVDs [25-34]. Raising public awareness of various health issues can lead to the implementation of preventive measures [35]. The guidelines [36] state that, in general, visual aids (eg, graphs or images presenting the risk estimation) improve the understanding of risk and are more understandable than providing information in the form of numbers intended to improve results [35-38]. There is currently no official app or computer-based valid screening test that a health professional can use to show patients their health status through images [39]. Visual analytics enables efficient analysis and understanding of large datasets in real time [40]. Visual analysis, which is incorporated in apps, is an effective technique for educating society. The results of measures can be presented to help adults increase motivation and successfully change their behavior or lifestyle [28,34]. So, the results of measures can be presented to help adults increase motivation and successfully change their behavior or lifestyle [28,34].

The objective of this study is to review and compare the usability of existing estimations of CVD risk apps using the mHealth App Usability Questionnaire (MAUQ). Rather than focusing on traditional usability aspects related to user interaction design, this study assesses how effectively these apps adhere to usability standards as defined by the MAUQ.

## Methods

This review used quantitative research methodology to review and compare the usability of existing estimations of CVD risk apps.

### Systematic Review of Apps for Estimation of CVD Risk

In March 2023, we reviewed apps for estimating CVD risk available in the Google Play Store [41] using the Samsung Galaxy Tab S6 Lite and Samsung Galaxy S8+ with Android operating systems. In addition, we reviewed apps in the Apple App Store [42] using the iPhone 13 Pro Max and iPad Pro (3rd generation) with iOS operating systems. The review of the apps was conducted by two researchers separately from each other. To ensure consistency, any discrepancies between the two reviewers were resolved through discussion and consultation with a third reviewer. Search strings including “CVD”, “CVD risk calculator”, “cardiovascular diseases”, and “cardiovascular risk” were used for searching the apps suitable for evaluation. According to the PRISMA (Preferred Reporting Items for Systematic Reviews and Meta-Analyses)(Checklist 1) [43] flow diagram, we searched for apps and excluded or included them based on their title, icon, and description in the app store.

The coding process categorized key aspects from all apps, names, and website links from each web store were manually transferred into Excel spreadsheets. The next step was to open and analyze each app on distribution platforms by looking into their titles, icons, screenshots or videos, and descriptions. Apps were included in the following steps if the title of the app is referenced to prognostic models for estimating CVD risk; the icon of the app represents some information about a possible way of estimating CVD risk; if the screenshots or video of the app gives an inside look at questions or results of prognostic models for estimation of the CVDs risk; if the description in the software platform provides information or references to a prognostic model for estimating CVD risk. Each relevant app was downloaded on a mobile phone or tablet in the final step. We have included free apps in the English language for various software platforms and their sections (Medical, Health and Wellness, Health and Fitness, etc), which estimate CVD risk and can be used either for personal (eg, estimating individual CVD risk in home environments) or for professional (eg, assessing patient CVD risk in clinical settings) purposes. Whether the app was developed for health care professionals or patients was determined according to the manufacturer’s intended use. Apps were excluded in case of duplicates (from the search string and distribution platforms), non-English language, technical issues, paid apps, and apps not related to estimating CVD risk and having different purposes (games, quizzes, journals, etc).

We also categorized apps according to whether they were single calculators containing one prognostic model for estimating CVD risk or multi-calculators containing more than one prognostic model for estimating CVD risk, as done by Fijačko et al [44]. Results were summarized with means and SDs. We also calculated the intraclass correlation coefficient (ICC_2,k_, intraclass correlation coefficient, 2-way random, average measures, and absolute agreement) [45-47].

### Evaluating the Usability of Apps for Estimation of CVD Risk

To measure the usability of existing apps designed to estimate CVD risk, we used the MAUQ [48]. MAUQ responses can be aggregated into (1) “Ease of Use” (8 items), (2) “Interface and Satisfaction” (6 items), and (3) “Usefulness” domains (7 items). All responses to the evaluating questions use a Likert scale, rated on a scale of 1-7 and higher scores mean better usability. The apps were evaluated by 3 experts with experience in health care informatics. The intraclass correlation coefficient (ICC_2,k_, intraclass correlation coefficient, 2-way random, average measures, and absolute agreement) [45-47] was calculated to represent the agreement of the app ratings.

### Statistical Analysis

Statistical analyses were conducted in October and November, 2023. Continuous variables were analyzed according to their Gaussian distribution and reported as mean with SD or 95% CI, whichever was appropriate. The data were analyzed using the SPSS statistical package version 29.0.0 (IBM Corp).

## Results

### App Selection

We identified a total of 2238 apps across both platforms. In the Google Play Store, we found 176 apps using the tablet and 376 apps using the phone. In the Apple App Store, we identified 802 apps on the tablet and 884 apps on the phone. In the next step, duplicates were removed (1175/2238, 53%), leaving 1063 apps for review. Then we excluded apps for several reasons: they were not available for free (10/1063, 1%), had inappropriate icons or names (280/1063, 26%), were game based (520/1063, 47%), had inappropriate descriptions (179/1063, 17%), or were not available in English (7/1063, 1%). This process narrowed the selection to 67 apps for further review. In the second phase, we installed and examined these 67 apps in detail, ultimately excluding those with incorrect content (49/67, 76%) and technical inadequacy (2/67, 3%). Incorrect content was identified based on criteria such as lack of evidence-based information, incompleteness, or lack of personalization features tailored to individual user profiles. Apps that did not provide a prognostic model for the estimation of risk or did not include critical risk factors (eg, blood pressure, cholesterol levels) were also considered poor content. Technical shortcomings were identified by several key factors, including app stability (frequent crashes or failure to load), usability problems (difficult navigation, nonintuitive interface), and compatibility problems (apps that did not work properly across different devices or operating systems). Apps with outdated or nonfunctional features were also excluded, as were those that caused security problems (eg, lack of data encryption for sensitive user information). As these factors have a significant impact on the reliability and user experience of apps, they were crucial in our elimination process.

**Figure 1.**
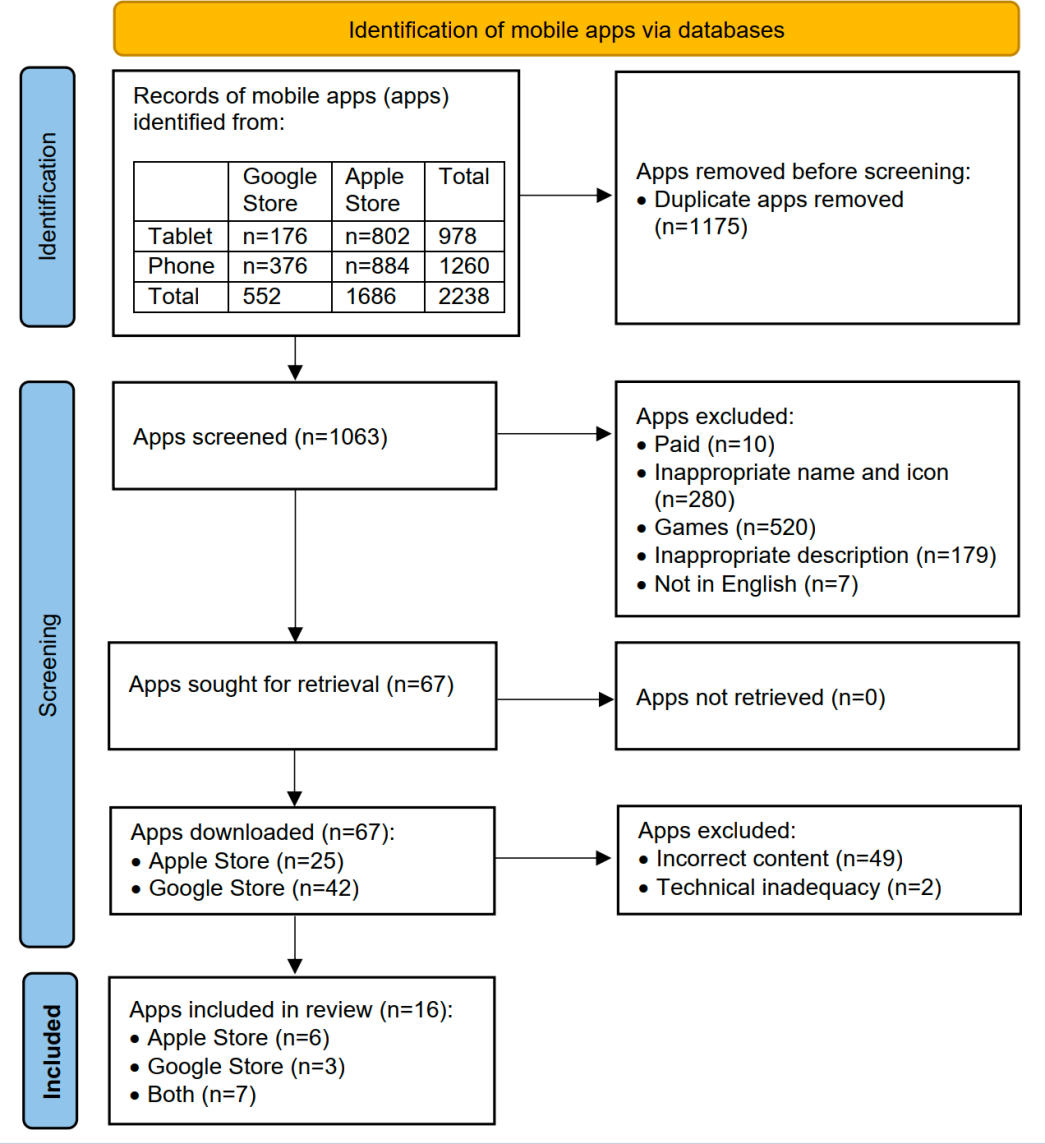
PRISMA flow diagram of the selection of included apps.

A total of 16 apps remained for analysis (Figure 1 [43]).

More characteristics of apps related to usability are presented in Multimedia Appendix 1.

Out of the 16 apps reviewed, 7 (44%) were compatible with both iOS and Android operating systems. Specifically, 4/16 (25%) apps were compatible with iOS, and 5/16 (31%) were compatible with Android. Compatibility refers to the ability of the apps to function on the respective operating systems without technical issues. Most of the apps (12/16, 75%) were designed for use by health care professionals in clinical settings as well as by individuals in home environments. A few apps (3/16, 19%) specifically indicated that they were intended for clinical use by health care professionals, including CV Risk Estimation, MediCalc (ScyMed, Inc), and CardioExpert I (Farid Belialov). In addition, one app (1/16, 6%), the Indigenous CVD Risk Calculator (An Tran Duy), was explicitly designed for use by individuals in home environments. The estimated results for CVD risk in apps were visualized in three formats across the apps: text, numerical, and graphical. Most apps (12/16, 75%) visualized results in both textual and numerical form. A smaller portion (4/16, 25%) presented CVD risk estimates in all 3 formats—text, numerical, and graphical—specifically in the following apps: ESC CVD Risk Calculation (European Society of Cardiology), Epi-RxlSK (University of Alberta), Cardiovascular Risk Calculator (www Machealth Pty Ltd), and Heartcare Lite (Heartcare Lite; Multimedia Appendix 2). All apps with graphical visualization were designed for use by health care professionals and patients (Table 1 and Multimedia Appendix 2).

**Table 1.** Descriptive characteristics of apps.

Name of apps	MOS^a^	Single (S^b^) or multi-calculators (M^c^)	Determination of use by developers (H^d^ and I^e^)	Visualization form of the cardiovascular disease (CVD) risk results
ESC CVD Risk Calculation	AND^f^ and iOS^g^	M	H/I	Text, numerical, graphical
CardioCal	AND and iOS	S	H/I	Text, numerical
CardioRisk Calculator	AND and iOS	S	H/I	Text, numerical
ASCVD^j^ Risk Estimator Plus	AND and iOS	S	H/I	Text, numerical
MediCalc	AND and iOS	M	H	Text, numerical
MDCalc Medical Calculator	AND and iOS	M	H/I	Text, numerical
Calculate by QxMD	AND and iOS	M	H/I	Text, numerical
CV Risk Estimation	iOS	M	H	Text, numerical
Indigenous CVD Risk Calculator	iOS	S	I	Text, numerical
Cardiovasculator Risk Calculator	iOS	S	H/I	Text, numerical, graphical
Epi-RxlSK	iOS	S	H/I	Text, numerical, graphical
Heartcare Lite	AND	S	H/I	Text, numerical, graphical
Framingham Score Heart Age	AND	S	H/I	Text, numerical
WHO^h^and ISH^i^ Cardiovascular risk prediction charts	AND	S	H/I	Text, numerical
CardioExpert I	AND	S	H	Text, numerical
ASCVD^j^ Risk	AND	S	H/I	Text, numerical

a MOS: mobile operating system.

b S: single calculator.

c M: multi-calculator.

d H: health professionals.

e I: individuals.

f AND: operation system for Android smartphones.

g iOS: Operation system for Apple smartphones.

h WHO: World Health Organization.

i ISH: International Society of Hypertension.

j ASCVD: atherosclerotic cardiovascular disease.

The reviewed apps feature 8 distinct prognostic models for estimating CVD risk, with the most common in all apps being the Framingham Risk Score (8/16, 50%), followed by the Atherosclerotic Cardiovascular Disease Risk model (7/16, 44%). Of the apps, 11/16 (69%) are single calculators using one prognostic model, while 5/16 (31%) are multi-calculators that incorporate multiple models. The multi-calculator app with the highest number of distinct prognostic models for estimating CVD risk was ESC CVD Risk Calculation, featuring 4 models (Table 2).

**Table 2.** Risk score method in apps.

Mobile apps	Single calculators (11/16, 69%)											Multi-calculators (5/16; 31%)					
Apps for estimating CVD riskPrognostic models (PM^a^) (n=8)	CardioCal	CardioRisk Calculator	ASCVD Risk Estimator Plus	Indigenous CVD Risk Calculator	Cardiovasculator Risk Calculator	Epi-RxlSK	Heartcare Lite	Framingham Score Heart Age	WHO^b^ and ISH^c^ Cardiovascular risk prediction charts	CardioExpert I	ASCVD Risk	ESC CVD Risk Calculation	MediCalc	MDCalc Medical Calculator	Calculate by QxMD	CV Risk Estimation	Total PM^a^^a^ in apps
Framingham Risk Score		✓			✓	✓		✓					✓	✓	✓	✓	8
Atherosclerotic Cardiovascular Disease Risk			✓								✓	✓	✓	✓	✓	✓	7
Reynolds Risk Score													✓	✓	✓		3
WHO^b^ and ISH^c^ risk prediction charts				✓			✓		✓								3
SCORE^d^ (Europe)										✓		✓					2
CVD^e^ Risk	✓																1
SMART risk score^f^												✓					1
ADVANCE^g^ risk score												✓					1
Total PM^a^:	1	1	1	1	1	1	1	1	1	1	1	4	3	3	3	2	26

a PM: prognostic models.

b WHO: World Health Organization.

c ISH: International Society of Hypertension.

d SCORE: Systematic Coronary Risk Evaluation.

e CVD: Cardiovascular disease.

f SMART risk score: which estimates 10-year risk of myocardial infarction (MI), stroke, or cardiovascular death.

g ADVANCE: the Action in Diabetes and Vascular disease: preterax and diamicron-MR controlled evaluation.

### Empirical Evaluation of Estimation of CVD Risk Apps

The overall average MAUQ score for all the apps was 5.6/7 (95% CI 4.33‐5.78). The app with the highest overall MAUQ score was MDCalc Medical Calculator 6.76 (0.25), followed by Heartcare Lite 6.58 (0.12), ASCVD Risk Estimator Plus 6.57 (0.18), ESC CVD Risk Calculation 6.11 (0.35), and Framingham Score Heart Age 6.03 (0.24). The app with the lowest MAUQ score was CardioRisk Calculator, 3.96 (0.21).

The “ease of use” domain had the highest overall average score across all domains (mean 6.20, SD 0.51). The apps with the highest overall MAUQ scores for ease of use were MDCalc Medical Calculator (mean 7, SD 0), followed by ESC CVD Risk Calculation (mean 6.92, SD 0.29), CardioCal (mean 6.73, SD 0.63), and Heartcare Lite (mean 6.60, SD 0.58). The lowest score in this domain was recorded for the CardioRisk Calculator (mean 5.27, SD 1.32).

The “interface and satisfaction” domain had the second highest overall average score (mean 5.25, SD 0.93). MDCalc Medical Calculator achieved the highest MAUQ score in this domain (mean 6.67, SD 0.33), followed by Heartcare Lite (mean 6.48, SD 0.79) and ASCVD Risk Estimator Plus (mean 6.24, SD 0.44). The lowest score in the “interface and satisfaction” domain was recorded by CardioRisk Calculator (mean 3.19, SD 1.13).

The “usefulness” domain received the lowest overall average score (mean 5.17, SD 1.13). The highest scores in this domain were achieved by ASCVD Risk Estimator Plus (mean 6.8, SD 0.32), followed by Heartcare Lite (mean 6.67, SD 0.58), MDCalc Medical Calculator (mean 6.60, SD 0.48), and Framingham Score Heart Age (mean 6, SD 0.32). The lowest score was recorded by MediCalc (mean 3.33, SD 0.47) (Table 3).

**Table 3.** mHealth App Usability Questionnaire mean scores.

Apps for estimating cardiovascular disease (CVD) risk	Ease of use, mean (SD)	Interface and satisfaction, mean (SD)	Usefulness, mean (SD)	Overall, mean (SD)^a^
ESC CVD Risk Calculation	6.92 (0.29)	5.95 (0.94)	5.47 (0.84)	6.11 (0.35)
CardioCal	6.73 (0.63)	5.48 (0.50)	4.07 (0.96)	5.43 (0.24)
CardioRisk Calculator	5.27 (1.32)	3.19 (1.13)	3.43 (0.89)	3.96 (0.21)
ASCVD^b^ Risk Estimator Plus	6.67 (0.67)	6.24 (0.44)	6.80 (0.32)	6.57 (0.18)
MediCalc	5.73 (0.75)	4.29 (0.81)	3.33 (0.47)	4.45 (0.18)
MDCalc Medical Calculator	7.00 (0.00)	6.67 (0.33)	6.60 (0.48)	6.76 (0.25)
Calculate by QxMD	6.47 (0.26)	5.62 (0.37)	4.93 (1.11)	5.67 (0.46)
CV Risk Estimation	5.60 (0.95)	4.29 (0.58)	3.60 (0.41)	4.50 (0.42)
Indigenous CVD Risk Calculator	5.80 (0.63)	4.24 (0.49)	4.33 (0.52)	4.79 (0.08)
Cardiovasculator Risk Calculator	6.07 (0.86)	5.24 (0.50)	5.33 (0.54)	5.55 (0.20)
Epi-RxlSK	6.13 (0.48)	5.52 (0.57)	5.8 (1.62)	5.82 (0.64)
Heartcare Lite	6.6 (0.58)	6.48 (0.79)	6.67 (0.58)	6.58 (0.12)
Framingham Score Heart Age	6.33 (0.52)	5.76 (0.80)	6 (0.32)	6.03 (0.24)
WHO^c^ and ISH^d^ Cardiovascular risk prediction charts	6.2 (0.58)	4.57 (0.80)	5.20 (0.42)	5.32 (0.19)
CardioExpert I	5.93 (0.45)	5.19 (0.35)	5.53 (0.71)	5.55 (0.18)
ASCVD^b^ Risk	5.73 (0.44)	5.29 (0.52)	5.6 (0.40)	5.54 (0.06)
Average score	6.2 (0.51)	5.25 (0.93)	5.17 (1.13)	5.60 (0.27)

a For the overall MAUQ score, we calculated reviewers inter-rater reliability using ICC, which showed excellent reliability (ICC_2,k_ 0.87; 95% CI 0.80‐0.91).

b ASCVD: atherosclerotic cardiovascular disease.

c WHO: World Health Organization.

d ISH: International Society of Hypertension.

## Discussion

### Principal Findings

Our review presented that apps for prognostic CVD risk estimation were available for both Android and iOS smartphones, designed for use by both health care professionals and patients. The most popular apps were single-calculator tools, each using only one CVD prognostic model. In contrast, the MAUQ highest-rated app, MDCalc Medical Calculator, offered access to multiple prognostic models, with the Framingham Risk Score being the most widely represented CVD prognostic model.

All apps were average quality, each achieving a high overall MAUQ score. In particular, the ’ease-of-use’ domain received the highest scores. More than half of the apps can be used by health care professionals or patients, but only a few offer more sophisticated visualizations for assessing estimation of CVD risk. Less than a quarter of apps included visualization. The apps that did include visualization were single calculators. In this article, we found considerable variation in the usability of apps for estimation of CVD risk, highlighting areas where developers can focus on improving the user experience. These findings are key to guide future improvements of mHealth, in terms of accessibility, user satisfaction, and overall functionality.

### Empirical Evaluation of Estimation of CVD Risk Apps

Our study assessed the effectiveness of these apps meeting usability standards as defined by the MAUQ of various apps designed to estimate CVD risk. Our findings revealed that most of the apps were predominantly characterized by positive feedback across multiple MAUQ questionnaire domains. In particular, the “ease-of-use” domain indicates a high level of practical acceptance and usability of the apps. Other studies have shown that the use of technology can promote shared decision-making by enabling health care professionals to manage chronic conditions [26,49-51]. The MAUQ evaluation tool has been used by several authors in different areas of health care. From ophthalmology (Australia) [52] to fitness (Malay) [53], breast cancer (German) [54], chatbot for reaching a patient (China) [55] etc. We also believe apps are an effective way to communicate the complex concept of clinical trials to patients and should also be incorporated in education curricula [37,56,57]. Based on the results, it can be concluded that the apps with the highest MAUQ scores are suitable for use by both health care professionals and patients. Rowland, et al [58] claim that despite the current limitations of diagnostic apps, there is a huge potential, and evidence is starting to emerge to demonstrate clinically significant improvements in morbidity and mortality outcomes in specific scenarios.

### Forms of CVD Risk Score Presented in Apps

Recently, there has been a growing interest in the use of visualization in digital humanities, which extends the way we interact with traditional information visualization methods and guides analytical processes. The main goal is to incorporate user feedback to improve automated analysis [59-63]. In our study, a few apps, such as ESC CVD Risk Calculation, Epi-RxlSK, Cardiovascular Risk Calculator, and Heartcare Lite, featured a visual risk display that helped to understand the risks of CVD to patients in a comprehensible manner. The Zolezzi et al [64] article also reported that participants found the EPI-RXISK app (University of Alberta) visually appealing, with a professional layout and use of simple technology. Visualization offers new capabilities to analyze health care systems and support better decision-making and patient motivation, but on the other hand, the presence of visualization does not automatically guarantee good usability, and an app without advanced visualization can still provide a good user experience [57,65,66].

From our review of various apps, it is evident that most patient-oriented apps incorporate visualization. Typically, these are single-function calculators and are predominantly available for free. Conversely, apps aimed at CVD risk estimation can assist patients in modifying risk factors and lifestyle habits, improving medication adherence, quality of life, and psychosocial well-being, and are associated with better outcomes in managing CVD risk factors [67,68] and lead to increased adherence to primary prevention strategies and reduced health care costs [69,70]. For health care professionals, the priority is to obtain results quickly and have access to multiple prognostic models and calculators within a single app [71-74]. Consequently, apps designed for estimating CVD risk not only deliver crucial information but also provide educational resources and guidance to health care professionals. They help with prevention, raise awareness, and encourage control of risk factors [71,72]. In addition, apps for estimation of CVD risk employing visualization to display data can increase patient motivation [73,74].

### Suggestions for Future Research

Electronic health record (EHR) systems are a digital representation of a patient’s paper-based medical documentation. They have emerged in recent years as a promising avenue for advancing clinical research [75-77]. In a recent study, the authors examined differences in improvements in hypertension guideline implementation using standard EHRs and EHRs that incorporated the use of apps with visual analytics dashboards for the estimation of CVD risk. They found that incorporating a specific app’s dashboard into EHRs has the potential to reduce the time and improve the implementation of hypertension guidelines [78]. The integration of apps with prognostic models for estimation of CVD risk directly into EHRs promises to be a valuable advancement for health care professionals [79-81]. This integration would allow the immediate estimation of CVD risk at the point of patient data entry, simplifying the process compared to using separate apps [82,83]. This efficiency could increase the effectiveness and quality of care management of CVD prevention strategies [84]. However, thorough evaluation and clinical testing are essential before apps can be seamlessly integrated into EHR systems and used in hospitals. In hospitals where the standard EHRs lack the capability to automatically calculate patient health risks, transitioning to EHRs with integrated apps with prognostic models would represent a substantial improvement in health care efficiency and accuracy. Even more, in the research by the author Serbanati [85] and other studies, they integrated AI into the EHRs and created an intelligent agent that acts as an avatar of the individual’s health. This agent collaborates with health care professionals, provides information on all aspects of an individual’s health, proactively offers solutions, and helps them diagnose and decide on the right treatment [79,86].

Apps for estimation of CVD risk should be designed based on prognostic models for effective use in health care. Other studies conclude that the Framingham Risk Score is the most widely used prognostic model for the estimation of CVD risk. We also found out that the most used prognostic model in analyzed apps was the Framingham Risk Score, and no AI prognostic models were used in any of the apps [49-51,87]. The authors of the review article reference findings indicating that 50% of their studies have already incorporated prognostic models for estimating CVD risk, developed using AI [88]. Estimation of CVD risk using AI in general and CVDs prediction specifically is becoming increasingly common, but it is crucial to ask critical questions before using these prognostic models [89].

### Limitations

Our study also has a few limitations. The first limitation is that we excluded paid apps, so we may have missed some quality apps that are maybe connected to EHRs or had prognostic models made by AI. The second limitation is that we focused only on usability and the inclusion of visualization in apps; we did not focus on other contextual analyses of the data, such as app usage in countries with the same Gross Domestic Product (GDP), apps for different races, disability levels, and so on. The latter could be a target for further research. The third limitation of the study is the apps industry, which is changing so rapidly that this type of review of apps is limited in time reliability. The fourth limitation is the number of MAUQ reviewers in this study and the representativeness of the target users’ evaluators. The future of integrating apps for estimation of CVD risk and EHR in the field of prevention of CVDs is very promising. Data collected through EHRs, apps, wearables, and remote monitoring systems will enable health care organizations to identify trends, risk factors, and patterns in cardiovascular outcomes [90].

### Conclusions

We presented the current state of apps for the estimation of CVD risk. We found that the most common prognostic model used in these apps was the Framingham Risk Score and that most of the apps were easy to use, indicating a high level of user satisfaction and acceptance. We discussed the benefits and challenges of using apps for the estimation of CVD risk in hospital settings by health care professionals or in home environments by patients. We suggested that apps for estimation of CVD risk be integrated into EHRs or other systems supported by health authorities to simplify the task of community risk estimation and improve the implementation of CVD prevention. However, we also acknowledged the need for further evaluation and testing of apps for estimation of CVD risk and prognostic models in clinical practice before they can be widely adopted and used in the hospital setting. We also argued that apps for estimation of CVD risk could provide patients valuable information, education, and guidance for prevention, as well as help modify risk factors and lifestyle habits, improve medication adherence, quality of life and psychosocial well-being, and reduce health care costs.

## Supplementary material

10.2196/56466 Multimedia Appendix 1 Additional information of apps.

10.2196/56466 Multimedia Appendix 2 Apps with visualization.

10.2196/56466 Checklist 1 Preferred Reporting Items for Systematic reviews and Meta-Analyses extension for Scoping Reviews (PRISMA-ScR) Checklist.

## Abbreviations

ADVANCE: Action in Diabetes and Vascular disease: preterAx and diamicroN-MR ControllEd evaluation
AI: artificial intelligence
CVD: cardiovascular disease
CVI: Content Validity Index
EHR: electronic health record
GDP: gross domestic product
I-CVI: Item-CVI
ICC_2k_: intraclass correlation coefficient
iOS: operation system for Apple smartphones
ISH: International Society of Hypertension
MAUQ: mHealth App Usability Questionnaire
mHealth: mobile health
MOS: Mobile Operating System
PM: Prognostic models
PRISMA: Preferred Reporting Items for Systematic Reviews and Meta-Analyses
QRISK: Cardiovascular Risk Score
S-CVI: Scale-level CVI
SCORE: Systematic Coronary Risk Evaluation
SCORE2: Systematic Coronary Risk Evaluation 2
SMART RISK score: which estimates 10 years risk of myocardial infarction (MI), stroke, or cardiovascular death
WHO: World Health Organization
