# Supplementary material for: Review and Comparative Evaluation of Mobile Apps for Cardiovascular Risk Estimation: Usability Evaluation Using mHealth App Usability Questionnaire
Source: JMIR Mhealth Uhealth. 2025 May 8;13:e56466. doi: 10.2196/56466 (PMC12080973; doi:10.2196/56466)
Supplement: Multimedia Appendix 1 [file mhealth-v13-e56466-s001.docx]

**Table 1. Additional characteristics**

| Name of apps | Developer | Language | Age interval | Population | Input data | Unit of measure | Save result in MA |
| --- | --- | --- | --- | --- | --- | --- | --- |
| ESC CVD Risk Calculation | European Society of Cardiology | English | 30+ | SCORE (Europe), ASCVD (North America) | Data entry, choice between options | mg/dL, mmol/l, mmHg | No |
| CardioCal | Pixeloide | English, Portugal, Spanish | no limit | Only for 6 regions of USA | Data entry, choice between options | mg/dL, mmol/l, mmHg (US/SI) | No |
| CardioRisk Calculator | The University of British Columbia | English | no limit | International | Data entry | mg/dL, mmol/l, mmHg | No |
| ASCVD Risk Estimator Plus | American College of Cardiology Foundation | English | 40-59 | USA | Data entry, choice between options | US and SI: mg/dL, mmol/l, mmHg: | No |
| MediCalc® | ScyMed Inc. | English and Spanish | 30-74 | International | Data entry, choice between options | mg/dL, mmol/l, mmHg: conventional & SI unit entries | No |
| MDCalc Medical Calculator | MD Aware, LLC | English | no limit | International | Data entry, choice between options | mg/dL, mmol/l, mmHg: conventional & SI unit entries | Yes |
| Calculate by QxMD | WebMD | English, French, German, Italian, Portugese, Spanish | 40-79 | International | Data entry | mg/dL, mmol/l, mmHg | Yes |
| CV Risk Estimation | United Health Services, Inc | English | 30+ | International | Data choice between options | num | No |
| Indigenous CVD Risk Calculator | An Tran-Duy | English | no limit | USA and Europe | Data entry, choice between options | mg/dL | No |
| Cardiovasculator Risk Calculator | WWW Machealth Pty Ltd | English | no limit | International | Data entry, choice between options | mg/dL, mmol/l, mmHg | Yes |
| Epi-RxlSK | University of Alberta | English, Albanian | no limit | International | Data entry, choice between options | mg/dL, mmol/l, mmHg | No |
| Heartcare Lite | Heartcare Lite | English | no limit | International | Data entry, choice between options | mg/dL, mmol/l, mmHg | Yes |
| Framingham Score Heart Age | Alpen Mobile | English | no limit | International | Data entry, choice between options | mg/dL, mmol/l, mmHg: conventional & SI unit entries | Yes |
| WHO/ISH Cardiovascular risk prediction charts | Marco Peroni | English | 40-79 | International | Data entry, choice between options | mg/dL, mmol/l, mmHg | No |
| CardioExpert I | Farid Belialov | Russian, English | no limit | USA and Europe | Data entry | mg/dL, mmol/l, mmHg | Yes |
| ASCVD Risk | ScyMed Inc. | English | 0-90 | International | Data entry | mg/dL, mmol/l, mmHg | No |
