# Supplementary figures and images for: Review and Comparative Evaluation of Mobile Apps for Cardiovascular Risk Estimation: Usability Evaluation Using mHealth App Usability Questionnaire
Source: JMIR Mhealth Uhealth. 2025 May 8;13:e56466. doi: 10.2196/56466 (PMC12080973; doi:10.2196/56466)

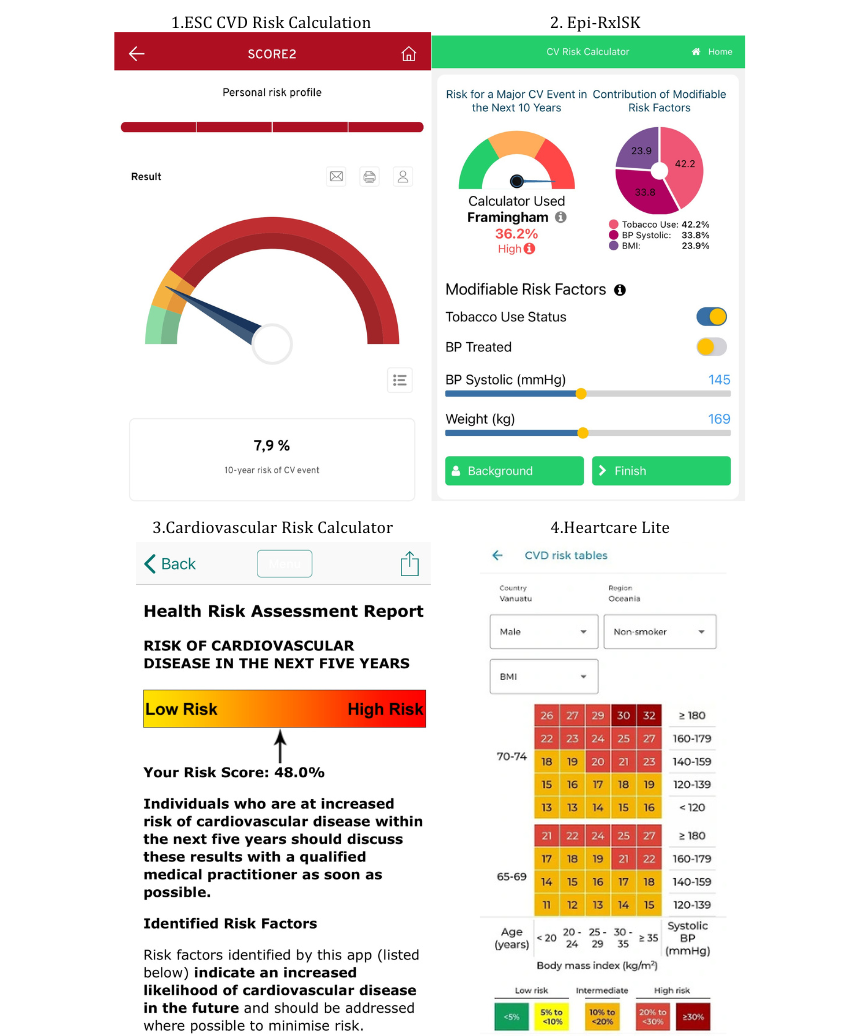

Supplement: Multimedia Appendix 2 [file mhealth-v13-e56466-s002.png]
